# A crucial role for B and T lymphocyte attenuator in preventing the development of CD4^+^ T cell-mediated herpetic stromal keratitis

**Published:** 2010-10-13

**Authors:** Likun Xia, Shengnan Zhang, Jiazi Zhou, Yan Li

**Affiliations:** Department of Ophthalmology, Shengjing Hospital, China Medical University, Shenyang,People’s Republic of China

## Abstract

**Purpose:**

To investigate the effect of the B and T lymphocyte attenuator (BTLA; CD272) on cluster of differentiation (CD)4^+^ T cell-mediated corneal immunopathology during murine herpetic stromal keratitis (HSK).

**Methods:**

BALB/c mice were infected with the herpes simplex virus type 1 (HSV-1) KOS strain by corneal scarification. The levels of BTLA expression in CD4^+^ and CD8^+^ T cells in murine peripheral blood were determined by flow cytometry on days 0, 3, 7, 10, 14, and 21 after HSV-1 infection. BTLA expression in the infected cornea was detected by immunohistochemistry. BALB/c mice were injected intraperitoneally with recombinant plasmid DNA encoding BTLA (pBTLA), pcDNA3.1, or PBS on 0 and 7 days before infection and 7 days postinfection. The incidence and severity of stromal disease, tear film virus titers, and the delayed-type hypersensitivity (DTH) reaction were then compared among treated and control groups. The effects of pBTLA on CD4^+^ T cells that infiltrated into infected corneas and on type 1 helper T-cell (Th1) cytokines (interferon-gamma [IFN-γ]) were evaluated. The levels of glycoprotein D (*gD*) mRNA in corneas were tested by real-time PCR. The eyes were examined histologically.

**Results:**

BTLA expression increased both in the corneas of HSV-1 infected mice and in CD4^+^ T cells in the murine peripheral blood. Systemic administration of pBTLA resulted in a diminished incidence and severity of corneal lesions compared to controls. Treatment with pBTLA led to a decreased infiltration of CD4^+^ T cells into infected corneas, and diminished Th1 responses in murine corneas, draining lymph nodes, and splenocytes. The pBTLA treated mice showed an impaired DTH response two weeks after HSV-1 infection compared to control mice. No differences were noted in tear film virus titers or *gD* mRNA levels in corneas among the experimental groups.

**Conclusions:**

The results suggest that recombinant pBTLA plays a crucial role in preventing HSV-1 specific responses in CD4^+^ Th1 cells in the infected corneas. Thus, BTLA, with immunosuppressive effects, may be a good candidate for treatment of HSK.

## Introduction

Corneal inflammation resulting from herpes simplex virus type 1 (HSV-1) infection of the eye results in herpetic stromal keratitis (HSK) that impairs vision and is a common cause of human blindness [1]. Studies in animal models of HSK have revealed that the corneal immunopathological lesions of HSK appear to be orchestrated mainly by cluster of differentiation(CD)4^+^ T cells that are primary type 1 helper T-cell (h1) cytokine producers [2-4]. Cytokines characteristic of Th1 cells (interferon-gamma [IFN-γ] and interleukin-2 [IL-2]) have been shown to dominate in HSK in preclinical and clinical phases of disease [5], and HSK can be abrogated by depletion of CD4^+^ T cells or by neutralization of Th1 cytokines [6,7]. Several studies have demonstrated that CD4^+^ Th1 cells require APC and co-stimulation to mediate HSK, and that blocking the 4–1BB/4–1BB ligand and B7/CD28 co-stimulatory interactions can each dramatically reduce inflammation in the infected cornea and prevent HSK [8-10].

The B and T lymphocyte attenuator (BTLA; CD272), a recently discovered co-receptor expressed in T cells, negatively regulates cell activation by recruiting phosphatase (SHP)-1/SHP-2, and shares structural and functional similarities with cytotoxic T-Lymphocyte Antigen-4 (CTLA-4) and programmed death 1(PD-1) [11]. Recently, the interaction partner of the BTLA herpes entry mediator (HVEM) has been identified as a member of the tumor necrosis factor receptor (TNFR) superfamily [12]. HVEM is predominantly expressed by resting T cells, monocytes, immature dendritic cells (DC), and endothelial cells [13,14]. BTLA is constitutively expressed by naïve CD4^+^ and CD8^+^ T cells and is further upregulated upon T cell activation [15]. It is also present in B cells, macrophages, and bone marrow-derived dendritic cells. Surface expression of BTLA and its accumulation at the immunological synapse are tightly regulated by T cell receptor ( TCR) and herpes entry mediator (HVEM) stimulation to deliver efficient inhibitory signals in the regulation of CD4^+^ T cell activation [16,17]. In accordance with the role of BTLA as a negative receptor, mice lacking the full-length form of BTLA are hyper-responsive to TCR-mediated activation of T cells [11]. BTLA or its ligand HVEM-deficient mice were more susceptible to immune and inflammatory diseases and showed more severe pathological tissue changes, such as experimental allergic encephalomyelitis, allergic airway inflammation, and intestinal inflammation, indicating that the BTLA pathway plays a critical role in immune-inflammatory disease [18-20].

In the present study, our aim was to analyze whether BTLA might impair development of HSK after HSV-1 infection of corneas of BALB/c mice. Our findings demonstrate that infection of BALB/c mice with HSV-1 KOS strain by corneal scarification resulted in upregulation of BTLA expression in the infected corneas and in CD4^+^ T cells from murine peripheral blood. Systemic administration of a recombinant plasmid DNA encoding BTLA decreased the numbers of CD4^+^ T cell that infiltrated into infected corneas, reduced Th1 response, impaired the delayed-type hypersensitivity (DTH) reaction, and abolished HSK lesion development. Our results are discussed in terms of novel approaches that merit testing for the control of HSK lesions.

## Methods

### Mice

Female BALB/c Mice, 5 to 7 weeks old, were purchased from the animal center of Beijing University (Beijing, China). All mice were maintained in a specific pathogen-free facility and were housed in micro-isolator cages containing sterilized feed, autoclaved bedding, and water. All experimental manipulations were undertaken in accordance with the institutional guidelines for the care and use of laboratory animals.

### HSV-1 Virus, corneal infection, and detection of ocular virus shedding

HSV-1 KOS strain was used for all procedures. A plaque-purified stock was grown and assayed on VERO cells in Dulbecco’s modified Eagle’s medium (DMEM), containing 5% fetal bovine serum, 100 U/ml penicillin, and 100 µg/ml streptomycin. Cells were cultured at 37 °C in a humidified incubator containing 5% CO_2_. The BALB/c mice were inoculated by the corneal route with HSV-1 strain KOS, as follows. Briefly, after the mouse was intraperitoneally anesthetized with 0.5% pentobarbital (45 mg/kg bodyweight), the corneal surface of the right eye was incised in a cross-shaped pattern with a sterile with a 27-gauge needle, and a 5 µl drop containing 1×10^5^ plaque-forming units (PFU) of HSV-1 KOS was applied to the eye and gently massaged with the eyelids. To detect ocular virus shedding after corneal HSV-1 inoculation, material from eye swabs was plated onto VERO cells, which were then monitored for cytopathic effects 48 and 96 h later.

### Flow cytometry analysis of BTLA expression on CD4^+^ and CD8^+^ T lymphocytes in murine peripheral blood after HSV-1 infection

On days 0, 3, 7, 10, 14, and 21 after corneal inoculation with HSV-1, murine peripheral blood was acquired and red blood cells in the cell suspension were removed using a lysing buffer (eBioscience, San Diego, CA). Before staining, single cells were washed and resuspended in staining buffer containing 1× PBS, 2%BSA, 10 mM EDTA, and 0.01 M NaN_3_. Cells were then blocked with an unconjugated anti-CD32/CD16 monoclonal antibody (mAb) for 15 min at 4 °C in staining buffer and stained by fluorescein isothiocyanate (FITC)-labeled anti-CD3e mAb, allophycocyanin (APC)-labeled anti-CD4 mAb or APC-labeled anti-CD8 mAb. Cells were then fixed and permeabilized using Cytofix/Cytoperm solution (eBiosciences) for 30 min, followed by washing with perm washing solution. Intracellular staining was done with phycoerythrin (PE)-labeled anti-mouse BTLA/CD272 mAb or PE-labeled mouse IgG1 isotype control for 30 min. All antibodies were purchased from eBiosciences Inc. Cells were incubated for 30 min on ice. After staining, the cells were washed twice with staining buffer, and samples were acquired on a FACSCalibur (BD Bioscience, San Jose, CA). Gates were set based on staining with the appropriate isotype control antibodies. The data were analyzed using the CellQuest software (BD Bioscience).

### Immunohistochemical detection of BTLA expression in murine corneas after HSV-1 infection

Enucleated eyes were collected at various time points post infection, fixed in 10% buffered neutral formalin, and embedded in paraffin. Sagittal sections 5 μm in thickness were then cut, deparaffinized, and rehydrated. Endogenous peroxidase activity was blocked by addition of 0.3% hydrogen peroxidase in methanol. Slides were boiled in a target retrieval solution buffer at high pressure for 2 min and then placed at room temperature (25 °C) for 20 min. The sections were then treated with normal goat serum and incubated overnight with rabbit anti-BTLA antibody (Santa Cruz Biotechnology, Santa Cruz, CA) at 1:200 at 4 °C. A biotinylated secondary antibody against rabbit IgG and an avidin-biotinylated peroxidase complex (Santa Cruz Biotechnology) were then used with 3,3′-diaminobenzidine (DAB) as a peroxidase substrate. Sections were counterstained with hematoxylin. Negative controls were performed by omitting the primary antibody.

### Plasmid pBTLA preparation

A 0.54 kb XbaI–HindIII fragment extracellular region of BTLA was PCR amplified using primers F: 5′-CAG TCT AGA GCC ACC ATG AAG ACA GTG CCT GCC ATG C-3′ (with XbaI site and initial code) and R: 5′-GTC AAG CTT TCA GCC TGG CCT CTC TTC CAT GGT G-3′ (with HindIII site and initial code) with a commercial clone A630002H24 (Shanghai GeneChem company, Shanghai, China) as a template. The PCR amplification reaction mixture (50 μl) consisted of dNTP (10 μmol/l; 1 μl), 10× advantage DNA polymerase2 buffer (5 μl), upstream and downstream primer, 1 μl each, DNA polymerase (1 μl), template DNA (1 μl), ddH2O (40 μl). Amplification was performed at 94 °C for 5 min, then 35 cycles at 94 °C for 30 s, at 55 °C for 30 s and at 72 °C for 2 min, followed by 10 min at 72 °C. The product was analyzed and purified by 1.0% agarose gel electrophoresis. The PCR amplified DNA fragments was inserted into pcDNA3.1(-) (Invitrogen, San Diego, CA) at the same restriction sites to create the expression vector pcDNA-BTLA (named pBTLA). Briefly, The pcDNA3.1(-) plasmid was obtained and purified according to the manufacturer’s instructions. The recycled plasmid segment and purified BTLA gene segment were recleaved with XbaI and HindIII respectively and purified by hyaline. The 10 μl connection system was obtained with the BTLA gene and pcDNA3.1 (BTLA:pcDNA3.1=1:1): pcDNA3.1 segment (4 μl), BTLA cDNA segment (4 μl), T4 DNA ligase (1 μl), 10× T4 DNA ligase buffer (1 μl), at 16 °C overnight. The recombinant was transformed into *E. coli* DH5a by the CaCl_2_ method and transformants were selected by agar plate containing aminopenicillin and identified by restriction enzyme digestion. Ten anti-aminopenicillin bacterial clones were chosen and shaken to amplify and distill the reconstruction plasmid, which was identified by cleaving with individual (XbaI or HindIII) and both (XbaI and HindIII) restriction enzymes. The sequence of the inserted fragment was confirmed by DNA sequencing (TaKaRa Biotechnology, Dalian, China).

The recombinant pBTLA was transfected into the HEK293 cell line (the Institute of Genetics of Fudan University, Shanghai, China) to express the target protein BTLA, which was detected by indirect immunofluorescence and western blotting. HEK293 cells were cultured in minimum essential medium (MEM) supplemented with MEM nonessential amino acids (Gibco, Grand Island, NY), 10% FBS, 110 µg/ml sodium pyruvate, and antibiotics (100 U/ml penicillin, 100 µg/ml streptomycin and 2.5 μg/ml M-Plasmocin). A total of 5×10^5^ cells were seeded into each well of a six-well plate. On the third day (when the cells had reached 80%–90% confluence), the culture medium was aspirated, and the cell monolayer was washed with pre-warmed sterile PBS. Cells were transfected with the recombinant pBTLA or the empty vector pcDNA3.1 using Lipofectamine 2000 reagent (Invitrogen) according to the manufacturer's instructions. Four micrograms of plasmid DNA (in 10 µl ddH2O) was mixed with 250 µl of serum-free medium (SFM) and incubated for 20 min at room temperature (25 °C) with a mixture of 10 µl Lipofectamine 2000 and 250 SFM and the resulting complex mixture was added to a monolayer of pre-confluent cells seeded in a six-well plate. Forty-eight hours after transfection, the cells were fixed with 100% acetone at –20 °C for 15 min and BTLA expression was detected using the indirect immunofluorescence assay, with a monoclonal antibody against the BTLA protein and a FITC-labeled rabbit-anti-mouse IgG (Sigma, St. Louis, MO).

For western blot analysis, at 24, 48, and 72 h after transfection, the transfected cells were washed twice with PBS (pH 7.4) and boiled for 10 min in lysis buffer (20 mM Tris–HCl, pH 7.6, 150 mM NaCl, 1% Nonidet P-40, 0.5% sodium deoxycholate, 0.1% sodium dodecyl sulfate). Cell lysates were analyzed by 12% sodium dodecyl sulfate PAGE (SDS–PAGE) and transferred onto nitrocellulose membrane, which was blocked by 5% skim milk. Primary antibodies, anti-BTLA protein mAb, and a HRP-labeled rabbit-anti-mouse IgG (1:3,000; Sigma) were diluted in washing buffer (120 mM NaCl, 10 mM Tris, pH 7.4, 0.1% Tween-20) and incubated for 1 h or 30 min at room temperature. After three PBS washes and one additional wash with PBS without Tween-20, proteins bound to the filter were scanned using an Odyssey infrared imaging system at 680- and 800-nm wavelengths.

### Plasmid pBTLA administration

The plasmids were purified and dissolved in DNase-free sterile water to concentration of 1 µg/µl. Mice were administrated intraperitoneally (i.p.) on days, −7, 0, and 7 with 100 µg of pBTLA or 100 µg of control plasmid containing pcDNA3.1, or 100 µl of phosphate buffered saline (PBS). Each group included fifteen animals. On day 0, the mice were scarified lightly on their cornea with a 27-gauge needle and infected with a 5 µl drop containing 1×10^5^ PFU of HSV-1 strain KOS.

### Virus titration

Swabs of the corneal surface were collected from day 0 to 7 after corneal inoculation. The swabs were put into sterile tubes containing 1 ml serum-free DMEM with 100 U/ml penicillin and 100 µg/ml streptomycin, and were stored at −80 °C. For detection and quantification of virus, the samples were thawed and swirled gently to distribute the sample. Individual subsamples (200 µl of each sample) were diluted further, and viral titers were determined by standard plaque assays [21]. Briefly, monolayer VERO cells were cultured in 12 wells multiwell plates and washed with Dulbecco's phosphate-buffered saline. Aliquots (200 µl) of virus dilutions were then added to wells. After incubation for 1 h at 37 °C, maintenance medium (DMEM containing 5% fetal bovine serum, 100 U/ml penicillin and 100 µg/ml streptomycin) was added, and the infected monolayers were incubated for additional 36 to 48 h to allow the formation of discrete, well formed plaques that could be counted. The cells were fixed and stained with 1% crystal violet dissolved in a 10% formalin solution, and the number of microscopic plaques was counted under a microscope.

### Clinical observations

The eyes were examined on different days after infection for the development of clinical lesions by slit-lamp biomicroscopy, and the clinical severity of stromal keratitis of individually scored mice was recorded. The stromal keratitis was graded on a scale of 0 to 4+ for corneal opacity, corneal neovascularization, edema, and infiltration: 0, not present; 1+, less than 25%; 2+, between 25% and 50%; 3+, between 50% and 75%; and 4+, between 75% and 100% [22].

### Delayed-type hypersensitivity (DTH) response

DTH responses were measured in uninfected (naïve) and infected mice, including treated and control groups, on day 13 after HSV-1 infection. Briefly, 1×10^5^ PFU of UV-inactivated HSV-1 KOS in 30 µl of DMEM was injected into the right rear footpad of each mouse. The left rear footpad was injected with the same amount of virus-free tissue culture medium. Footpad swelling was measured with a micrometer immediately before and 24 h after injection. HSV-specific footpad swelling was determined by the formula: [right footpad swelling at 24 h-right footpad swelling before injection]-[left footpad swelling at 24 h-left footpad swelling before injection] [23].

### Isolation of infiltrating cells in the corneal stroma and draining lymph nodes (DLNs) and flow cytometry

Single-cell suspensions were prepared from corneas and cervical DLNs on day 14 postinfection. For flow cytometry measurement of the infiltrating cells of the cornea, six corneas per group were collected at the indicated time points by dissecting the corneal buttons above the limbus with a scalpel. The pooled corneas were incubated with 60 U/ml Liberase (Roche Diagnostics Inc., Indianapolis, IN) for 60 min at 37 °C in a humidified atmosphere of 5% CO_2_. After incubation, the corneas were disrupted by grinding with a syringe plunger on a stainless steel mesh and a single-cell suspension was made in RPMI 1640 medium with 10% FBS. Cells were counted by trypan blue exclusion for high viability. Next, the single-cell suspensions obtained from corneal or lymphoid samples were stained for flow cytometry. Before staining, cells were washed and resuspended in staining buffer containing 1×PBS, 2%BSA, 10 mM EDTA, and 0.01 NaN_3_. Cells were then blocked with an unconjugated anti-CD32/CD16 mAb for 15 min at 4 °C in staining buffer and stained by fluorescein isothiocyanate (FITC)-labeled anti-CD3e mAb, allophycocyanin (APC)-labeled anti-CD4 mAb. Next, cells were fixed and permeabilized using Cytofix/Cytoperm solution (eBiosciences) for 30 min, followed by washing with perm washing solution. Intracellular staining was done with phycoerythrin (PE)-labeled anti-mouse IFN-γ mAb for 30 min. PE rat IgG1 was isotype control for anti-IFN-γ antibodies. All antibodies were purchased from eBiosciences Inc. Cells were incubated for 30 min on ice. After staining, the cells were washed twice with staining buffer, and samples were acquired on a FACSCalibur (BD Bioscience). Gates were set based on staining with the appropriate isotype control antibodies. The data were analyzed using the CellQuest software (BD Bioscience).

### IFN-γ assay by enzyme-linked immunosorbent assay (ELISA)

On day 14 postinfection, splenocytes were harvested from the mice from the different groups using Ficoll-Hypaque density gradient centrifugation. Cells were resuspended at a density of 1×10^6^ cells/ml in RPMI 1640 medium containing L-glutamine (2 mM), penicillin/streptomycin (100 U/ml), and 10% fetal calf serum. Cells were cultured in 96-well plates with UV-inactivated HSV-1 strain KOS (Multiplicity Of Infection=1.5 before UV inactivation). Con A-stimulated (5 µg/10^6^ cells/ml) and unstimulated cells were used as positive and negative controls, respectively. Plates were incubated at 37 °C for 72 h. The supernatant fluid was collected and stored at −80 °C until use. These supernatants were screened for the presence of IFN-γ by an ELISA development kit (R&D Systems, Minneapolis, MN) according to the manufacturer's instructions, with a detection limit of 5 pg/ml for IFN-γ.

### Real-time quantitative PCR analysis

Total RNA was obtained (RNeasy Mini kit; Qiagen Co, Hilden, Germany) from infected corneas in different treatment groups at 72 h after HSV-1 infection and purified (on-column RNase-free DNase kit; Qiagen Co). RNA (1 µg) was reverse transcribed with 10 ng/ml oligo(dT) and SuperScript^TM^ III reverse transcriptase (Invitrogen) following the manufacturer’s protocol. Amplification was performed at 65 °C for 1 min, at 30~65 °C for 30 min, at 60 °C for 30 min, at 98 °C for 5 min, then at 5 °C for 5 min. The cDNA obtained was used as a template for real-time quantitative PCR. The specific primers used were murine *gD* forward (5′-GGC GAA GCT CAC TAC TCA C-3′) and reverse (5′-GCG TCC ATA GCG TCC T-3′). Reactions were set up with cDNA corresponding to 100 ng total RNA, primers (0.4 µmol) and 10 μl SYBR green PCR master mix (TaKaRa Co, Shiga, Japan) made to a total volume of 20 µl with nuclease-free dH_2_O. The iQ5 real-time PCR detection system (Bio-Rad, Foster, CA) was used to determine the levels of SYBR green fluorescence 45 cycles. Samples were degeneration for 10 min at 95 °C, anneal at 57 °C for 20 s, with extension of the primer product at 72 °C for 15 s, 45 cycles then a final 72 °C step for 20 s that removes background fluorescence from primer-dimers. A melt curve analysis was produced at the end of the cycling to ensure the specificity of PCR product amplification and associated SYBR green fluorescence. Initially, mRNA levels were normalized to the *GAPDH* mRNA levels. The *gD* expression relative to *GAPDH* was analyzed by the 2–ΔΔCT method [24]. A relative change in expression of twofold was set as a threshold for determining whether differential expression of a gene had occurred. The p values associated with the fold-change in expression were calculated using a one-way ANOVA (ANOVA) on the real-time PCR Ct values among different groups.

### Histopathology

Animals were euthanized on day 14 postinfection. The eyes were removed and fixed in 4% formaldehyde, dehydrated, embedded in paraffin, and cut into 5-µm sections. The sections were stained with hematoxylin and eosin (HE) according to a standard protocol, and examined by light microscopy (Olympus, Tokyo, Japan).

### Statistical analysis

All experiments were performed twice. Mean±standard deviation (SD) values were calculated. A one-way ANOVA (ANOVA) was used to determine the differences among groups with respect to the numbers of CD4^+^ and CD4^+^ IFN-γ^+^ T cells, cytokine assay, plaque assays, DTH, *gD* mRNA levels, clinical findings, and followed by a Fisher’s LSD test. A χ^2^ test was used to evaluate the incidence of disease. A p<0.05 was regarded as a significant difference among groups.

## Results

### BTLA expression is upregulated in the murine peripheral blood and cornea after HSV-1 infection

**Figure 1:**
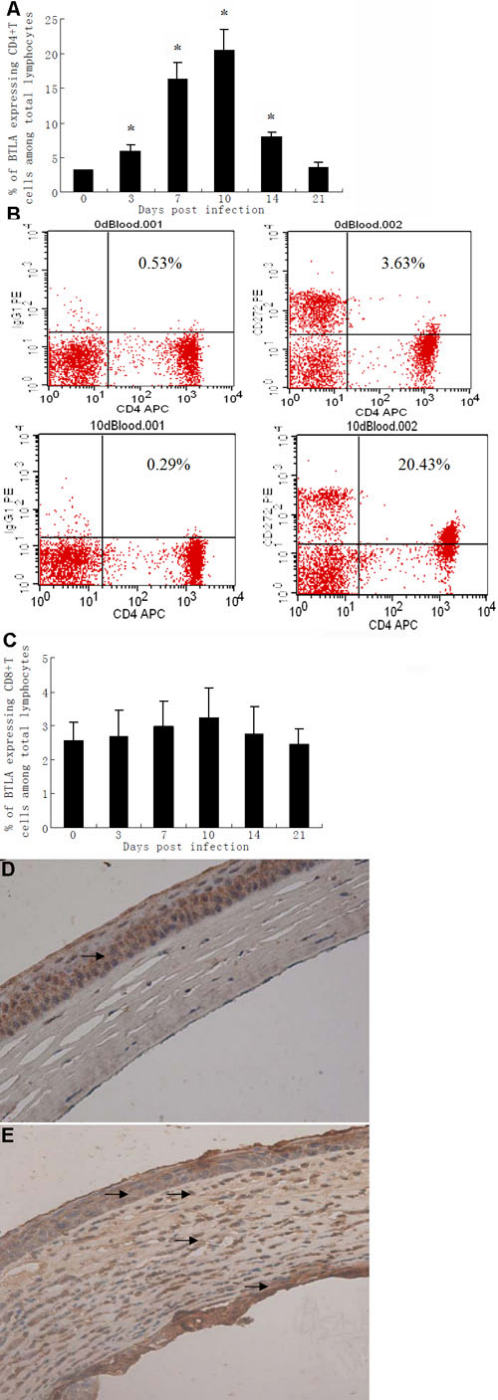
BTLA expression is upregulated in murine peripheral blood and corneas after HSV-1 infection. The expression of BTLA on CD4^+^ and CD8^+^ T lymphocytes in murine peripheral blood was determined by flow cytometry on day 0, 3, 7, 10, 14, and 21 after corneal inoculation with HSV-1 (n=6 at each time point). Peripheral blood mononuclear cells (PBMC) were stained simultaneously for CD3, CD4 or CD8, and BTLA. PE mouse IgG1 was used as isotype control. **A**: The percentage of CD4^+^ T cells expressing BTLA at the indicated time point is shown in the bar diagrams. Statistically significant differences were observed on day 3, 7, 10, and 14 compared to day 0 (*p<0.01). **B**: Representative three-color flow cytometry plot of pre-infection (day 0) versus day 10 post-infection, gated on CD4^+^ T cells indicating BTLA expression in murine peripheral blood. **C**: The percentage of CD8^+^ T cells expressing BTLA for the indicated time point is shown in the bar diagrams. No statistically differences were observed on day 3, 7, 10, 14 and 21 compared to day 0 (p>0.05). Corneal tissues (**D**,**E**) were analyzed by immunohistochemistry to determine BTLA expression. Immunohistochemical staining with an antibody against BTLA was performed to detect BTLA expression. Sections incubated without a primary antibody served as negative controls. All tissue sections were counterstained with hematoxylin. Brown staining indicates BTLA expression. **D**: Corneal tissues derived from a normal uninfected mouse. Faint BTLA expression was only observed in the corneal epithelium (arrow). **E**: Cornea tissue derived from a mouse 10 days after corneal HSV-1 inoculation. Increased BTLA expression was observed in the corneal epithelium, stroma, endothelium, and many inflammatory cells that infiltrated the corneal stroma (arrows). Original magnifications, 400×.

To confirm whether BTLA takes part in the immunopathogenesis of HSK in BALB/c mice, we first tested the expression of BTLA in the cornea and peripheral blood of HSV-1 infected mice. BTLA expression increased in CD4^+^ T cells, peaking on day 10 after infection (Figure 1A). A representative plot gated on CD4^+^ T cells demonstrated higher BTLA expression (20.14%) on day 10 after corneal inoculation with HSV-1 compared to day 0 (3.10%, Figure 1B). However, BTLA expression did not increase in CD8^+^ T cells after HSV-1 infection (Figure 1C).

Immunohistochemistry results for BTLA expression in the corneas are shown in Figure 1D. In the normal uninfected mouse cornea, BTLA expression was only detected in the corneal epithelium (Figure 1D). However, in corneal tissues derived from HSV-1 infected mice, BTLA expression was increased in the corneal epithelium, stroma, and endothelium (Figure 1E). In addition, the stroma of infected cornea contained many inflammatory cells that expressed BTLA (Figure 1E). These results indicate that BTLA takes part in the immunopathogenesis of HSK in BALB/c mice.

### Identification of recombinant plasmid pBTLA

**Figure 2:**
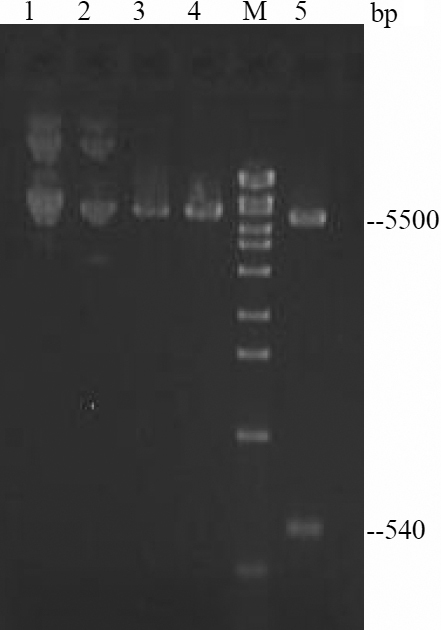
Restriction enzyme digestion of recombinant plasmid pBTLA. M: DNA marker. 1: pBTLA 2: pcDNA3.1 3: pBTLA/HindIII 4: pBTLA/XbaI 5: pBTLA/HindIII+XbaI.

Recombinant plasmids were digested to 540 bp and 5,500 bp DNA fragments, which carried the target genes *BTLA* and pcDNA3.1 (Figure 2). Analysis of DNA sequencing showed the amplified *BTLA* gene was identical with that listed in GenBank. The restriction endonuclease digestion and sequence analyses confirmed successful construction of the recombinant expression plasmid pcDNA-BTLA (named pBTLA).

### Expression level of the recombinant plasmid pBTLA

**Figure 3:**
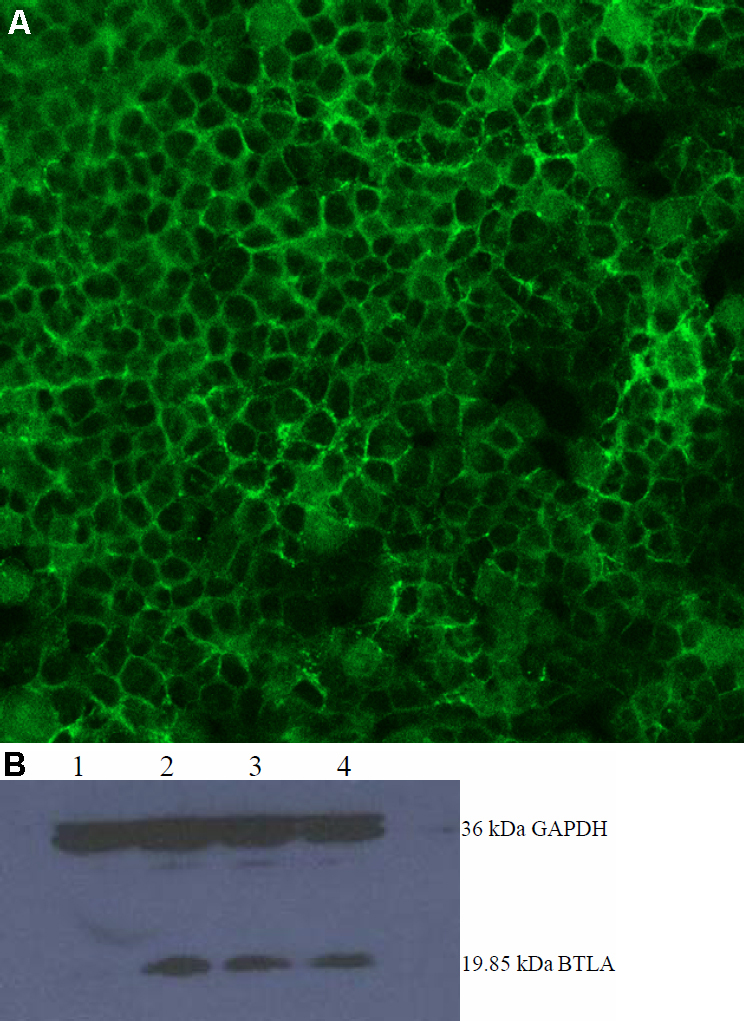
Expression level of the recombinant plasmid pBTLA. **A**: Indirect immunofluorescence detection of BTLA in HEK293 cells. HEK293 cells were previously transfected with 1 μg of the recombinant plasmids pBTLA or the empty vector, pcDNA3.1, in a 12-well plate. Forty-eight hours post-transfection, cells were fixed with acetone, treated with anti-BTLA monoclonal antibody (mAb), and stained with FITC conjugated secondary antibody. Green: BTLA protein-positive HEK293 cells transfected with pBTLA in fluorescence microscope. **B**: western blot result of recombinant plasmid pBTLA. HEK293 cells were transiently transfected with 1 μg of pBTLA. Cells were harvested at 24, 48, 72 h after transfection, lysed, and the cell extract was resolved on a 12% SDS–PAGE gel. Protein was transferred to a PVDF membrane (Bio-Rad) using a semi-dry transfer apparatus. Western blot membranes were scanned using an Odyssey infrared imaging system (Trans-Blot SD, Bio-Rad). Lane 1 stands for the pcDNA3.1 transfected cell extracts, lanes 2–4 stand for the pBTLA transfected cell extracts at 24, 48, and 72 h post-transfection.

As shown in Figure 3A, at 48 h post-transfection, the immunofluorescence intensity was high in HEK293 cells transfected with pBTLA. No fluorescence was observed in the pcDNA3.1 transfected cells using anti-BTLA protein-specific mAb.

As shown in Figure 3B, western blotting revealed a band in the pBTLA transfected cell extracts (lanes 2–4) at approximately 20 kDa size (our target BTLA protein 19.85 kDa). In the pcDNA3.1 transfected cell extracts (Figure 3B; lane 1), no 19.85 kDa band was found. The results indicate that the target protein BTLA was expressed successfully.

### Treatment with pBTLA decreases ocular disease severity

**Figure 4:**
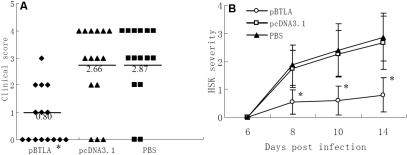
Treatment with pBTLA decreases the clinical severity of HSK. HSK lesion severity was determined by slit-lamp biomicroscopy, and the clinical severity of stromal keratitis of individually scored mice was recorded. Stromal keratitis was graded from 0 to 4+, depending on the corneal opacity with neovascularization, edema, and infiltration. Each group of mice consisted of fifteen animals, and the results shown are the representative of two similar experiments. A. Each dot represents corneal opacity of an individual BALB/c eye on day 14 following HSV-1 infection. Crossbar indicates the mean. *p<0.01 versus control groups. B. HSK lesion scores in three groups of mice at different time points post infection. *p<0.01 versus control groups.

Following infection of the cornea with HSV-1, virus replicated in the epithelium and damaged this tissue. Repair occurred rapidly, and virus was no longer recovered in tear films after 6 days. At this time, inflammatory responses in the cornea were also not grossly evident. However, beginning at 7 to 8 days after infection, opacities of the cornea became evident and persisted. Mice that were treated with pcDNA3.1 or PBS progressed to severe inflammation, with nearly 85% of infected corneas progressing to at least severity score 3. On day 14 after infection, 12 of 15 pcDNA3.1 treated eyes showed severe corneal opacities, and 10 eyes were considered as scoring over 3. In all, 13 of 15 PBS treated eyes developed severe corneal opacities, and 11 eyes showed scores above 3. In contrast, treatment of mice with pBTLA showed markedly milder stromal keratitis, with only 7 of 15 pBTLA treated eyes developing mild corneal opacities, and only one progressing to severity score 3 (Figure 4A). A comparison of average severity scores at days 8, 10, and 14 postinfection showed these to be significantly lower (p<0.01) in the pBTLA treated than the control groups (Figure 4B). These results indicated that pBTLA treatment decreases ocular disease severity.

### Treatment with pBTLA reduces CD4^+^ T cell response to HSV-1 infection in corneas

**Figure 5:**
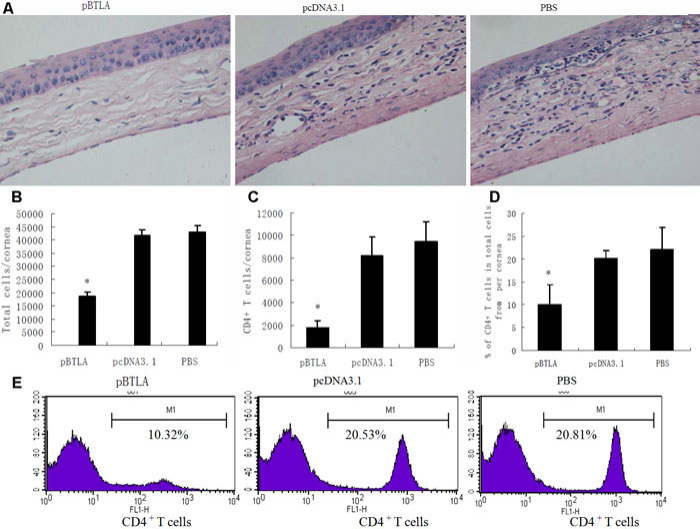
Decreased inflammation in the corneas of mice treated with pBTLA. **A**: pBTLA treated, pcDNA3.1 treated and PBS control mice were infected with 1×10^5^ PFU of HSV-1 KOS. On day 14 postinfection, mice were euthanized, and eyes were processed for cryosectioning. H&E staining was conducted on 5-µm sections. The corneas in the pcDNA3.1 treated and PBS control groups show severe swelling, heavy infiltration with inflammatory cells, and numerous neovascular structures in the stroma. The cornea in the pBTLA treated group exhibits only mild stromal edema, little inflammatory cell infiltration, and fewer neovascular structures. Original magnification, 400×. **B**: Six corneal samples from each group of animals were digested with liberase on day 14 post infection. The bars represent the total number of viable cells present per cornea of different groups of mice. *p<0.01 compared with control groups. **C**: The cells isolated from infected corneas were stained for CD4 marker and the bars represent the total number of CD4^+^ T cells present per cornea from different groups of mice. Decreased numbers of CD4^+^ T cells in the cornea of pBTLA treated mice. *p<0.01 compared with control groups. **D**: The percentage of CD4^+^ T cells in total cells of per cornea from different groups of mice. *p<0.01 compared with control groups. **E**: Representative plot shows CD4^+^ T cells expression on cornea of different group mice.

At 14 days postinfection, histological examination showed that the inflamed corneas of the pcDNA3.1 or PBS control mice were severely swollen, heavily infiltrated with inflammatory cells, and had numerous neovascular tissues in the stroma. In contrast, the corneas of pBTLA treated mice appeared to have only mild stromal edema and little inflammatory cell infiltration (Figure 5A,B). Single cell suspensions were prepared from corneas and analyzed by flow cytometry for CD4^+^ T cells numbers. As shown in Figure 5C-E, the absolute number and percentage of CD4^+^ T cells from cornea of pBTLA treated mice were significantly lower than those of pcDNA3.1 or PBS control mice (p<0.01). These results indicated that treatment with pBTLA dramatically reduced the CD4^+^ T cell response to HSV-1 infection in the cornea.

### The pBTLA treated mice showed a lower Th1 immune response

**Figure 6:**
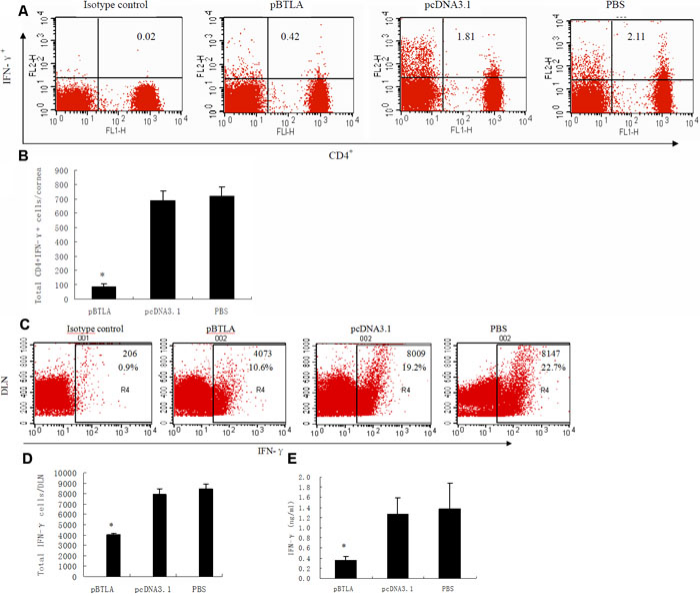
pBTLA treated mice show a lower Th1 immune response. **A**: On day 14 after HSV-1 infection, single-cell suspensions of cornea cells were prepared from mice from different groups. Cells were stained by anti-CD3e-FITC, anti-CD4-APC, PE anti-mouse IFN-γ- or PE Rat IgG1 Isotype control as described in Methods. Dot plots represent the frequencies of CD4^+^ IFN-γ^+^ T cells from pBTLA treated mice, pcDNA3.1 treated mice, and PBS control mice when gated on the lymphocytes. The percentage of CD4^+^ T cells expressing IFN-γ is shown in the upper right quadrants. **B**: The bar diagram demonstrates the total number of CD4^+^ IFN-γ^+^ T cells in the cornea in different groups (n=6 in each group). Decreased numbers of CD4^+^ INF-γ^+^ T cells are noted in the corneas of pBTLA treated mice (*p<0.01 compared with control groups). **C**: On day 14 after HSV-1 infection, single-cell suspensions of cervical draining lymph nodes were prepared from mice from different groups. Cells were stained by anti-CD3e-FITC, anti-CD4-APC, PE anti-mouse IFN-γ-, or PE Rat IgG1 Isotype control. Dot plots represent the frequencies of IFN-γ^+^ T cells from different group mice. The number of IFN-γ^+^ T cells and the percentage of IFN-γ^+^ T cells in all T cells are shown in the right quadrants. **D**: The bar diagram demonstrates the total number of IFN-γ^+^ T cells in the DLNs in different groups (n=6 in each group). Fewer numbers of IFN-γ^+^ T cells in the DLN of pBTLA treated mice (*p<0.01 compared with control groups). **E**: On day 14 after HSV-1 infection, spleen cells of the mice from different groups (n=6 in each group) were used in antigen specific ELISA. Levels of IFN-γ in pBTLA treated mice were lower than in pcDNA3.1 or PBS treated mice (*p<0.01).

In an attempt to explain why pBTLA treated mice showed slighter responses than those of pcDNA3.1 or PBS treated mice, we examined the number of CD4^+^ IFN-γ^+^ T cells in the corneas of different group mice on day 14 postinfection. Significantly fewer CD4^+^ IFN-γ^+^ T cells (p<0.01) were found in the cornea of pBTLA treated mice when compared with control groups (Figure 6A,B). Flow cytometry measurement also revealed fewer IFN-γ^+^ T cells in the draining lymph nodes (DLNs) of pBTLA treated mice (p<0.01) than in the control groups (Figure 6C,D). Splenocytes from pBTLA treated mice also produced significantly lower (p<0.01) amounts of IFN-γ as compared to pcDNA3.1 or PBS treated mice (Figure 6E). Thus, pBTLA treatment appeared to decrease the Th1 type of immune response of mice.

### Treatment with pBTLA suppresses the systemic DTH responses of mice

**Figure 7:**
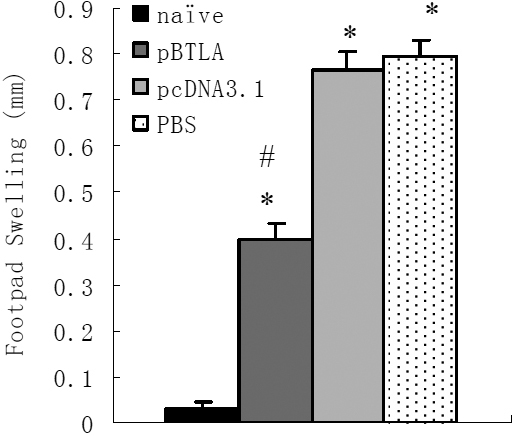
pBTLA plasmid treatment suppresses the systemic DTH responses of mice. pBTLA and pcDNA3.1 treated mice received 100 µg of the respective plasmid on days −7, 0, 7 relative to HSV-1 corneal inoculation, PBS treated mice received 100 µl PBS i.p. Two weeks after viral infection, DTH responses were measured in the footpad. All infected groups (n=6 in each group) had significant responses compared with the uninfected control mice (*p<0.01). DTH responses in pBTLA treated mice were lower than in pcDNA3.1 or PBS control mice (^#^p<0.01).

The DTH responses of the footpad in the different mice groups two weeks after viral infection are shown in Figure 7. The DTH responses in all infected groups were significantly higher than uninfected controls (p<0.01). In addition, DTH responses in pBTLA treated mice were lower than in pcDNA3.1 or PBS treated mice (p<0.01). The results indicated that pBTLA treatment inhibited immune cell-mediated inflammation induced by HSV-1.

### Treatment with pBTLA does not affect virus titers in tear film

**Figure 8:**
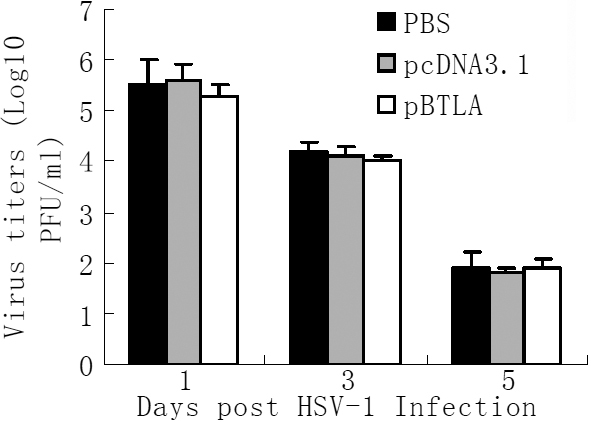
pBTLA treatment does not affect virus titers in tear film. Tear film virus titers were compared in the different mice groups following HSV-1 infection. Mice treated with pBTLA and pcDNA3.1 received 100 μg of the respective plasmid on days, −7, 0, 7 relative to HSV-1 corneal inoculation, and the PBS group received 100 µl PBS intraperitoneally. On days 1, 3, and 5, swabs of the corneal surface were collected and transferred to serum-free DMEM, and viral titers were determined by a plaque assay performed on VERO cells as described in methods. The virus titers was calculated as log_10_PFU/ml. Results are expressed as means±SD for individual eye swabs (n=6). No statistically significant differences were observed among the different groups (p>0.05).

Tear film virus titers on days 1, 3, and 5 following HSV-1 infection are shown in Figure 8. Mice systemically administered pBTLA showed no difference in virus titer from pcDNA3.1 treated or PBS control mice (p>0.05). Systemic administration of recombinant pBTLA therefore did not appear to affect virus titers in tear film.

### Treatment with pBTLA does not affect *gD* expression in cornea

**Figure 9:**
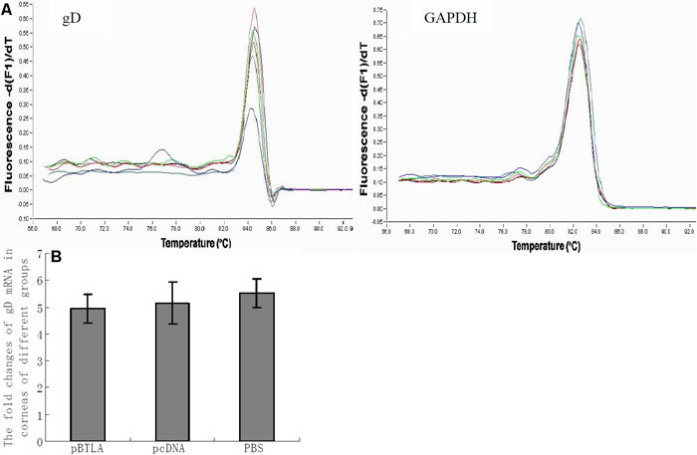
Effect of recombinant pBTLA on the levels of *gD* mRNA expression in cornea. Real-time quantitative PCR analysis of *gD* mRNA levels results are shown for corneas from the different mouse groups (n=6 in each group) at 72 h after HSV-1 infection. RNA was extracted from corneas of mice from the different groups and the levels of *gD* expression were evaluated through real-time quantitative PCR. **A**: The melt curves of *gD* and *GAPDH* after amplification in corneal samples. **B**: The fold changes of *gD* mRNA expression in corneas of different groups at 72 h postinfection. The *gD* mRNA levels in the corneas of pBTLA treated mice did not differ from those of the pcDNA or PBS control mice (p>0.05).

The *gD* mRNA levels in corneas from mice treated intraperitoneally with pBTLA and in, pcDNA3.1 treated mice and PBS control mice at 72 h after HSV-1 infection are shown in Figure 9. No differences were seen in levels of gD mRNA expression in the corneas of pBTLA treated mice compared to the control mice (p>0.05). These data indicated that systemic administration of recombinant pBTLA did not affect *gD* expression in the mouse cornea.

## Discussion

Herpes simplex virus type 1 infection of the mouse cornea leads to a transient epithelial lesion caused by HSV-1 replicating in and destroying corneal epithelial cells. This lesion is evident by 2 days after infection. Repair occurs rapidly, and virus characteristically can no longer be recovered in tear film after 5 to 6 days, at which time virus replication in the cornea has usually ceased. This means that after this time, the virus is thought to be expelled from corneal tissues by one or more aspects of host defense. However, T-cell invasion of the cornea begins 7 days after infection, a mild haze develops in the infected mouse cornea stroma, and limbal blood vessels begin to invade this normally avascular tissue. This represents the initiation of the chronic phase of the disease. The inflammation is progressive, leading to severe neovascularization and opacity, referred to as HSK [4].

Therapeutic targets in HSK include the HSV virus itself and the key host processes involved in the pathogenesis (the inflammatory immune response and angiogenesis). Treatment of HSK traditionally involves antiviral drugs and anti-inflammatory drugs such as corticosteroids. Development of an effective vaccine against HSV has proven to be elusive so far [4]. New information about immunological mechanisms would therefore provide novel avenues for the improvement of HSK therapy. The pathogenesis of HSK, as studied in the mouse, involves numerous components, but CD4^+^ T cells appear to be the principal orchestrators of the immunopathology [1-7,25,26].

The response of immune cells, particularly T cells and antigen-presenting cells (APCs), is dictated by the balance between negative signals from co-inhibitory receptors and positive signals from costimulatory receptors. Since CD4^+^ T cells are the primary effectors of stromal lesions of HSK, targeting costimulatory and coinhibitory signals of CD4^+^ T cell activation and decreasing the numbers of CD4^+^ T cells would be a useful therapeutic approach [8-10,27]. The best-characterized costimulatory signals determined to be important in the initiation of the immune response are the B7/CD28 and CD40/CD154 receptor-ligand interactions. Several groups have studied the roles of these two costimulatory signals in viral pathogenesis by exploring the effects of blocking costimulatory signals in the well characterized lymphocytic choriomeningitis virus (LCMV) and vesicular stomatitis virus (VSV) models in mice. Antiviral CD4^+^ T-cell responses to both viruses are moderately dependent on these costimulatory signals [28].

The B7/CD28 and CD40/CD154 costimulatory signals have also been successfully targeted for therapeutic intervention in primary HSV infection [8,9,29]. Systemic blockade of B7/CD28 costimulatory interactions using fusion protein CTLA4Ig minimizes the immunoinflammatory lesions caused by HSV infection of the cornea [8,9]. Disruption of B7/CD28 and CD40/CD154 interactions with blocking reagents (CTLA4Ig and MRI) or in mice with genetic deficits in either CD28 or CD154, profoundly impaired CD4^+^ T-cell responses during primary acute HSV-1 infection of the hind footpads [29]. Another candidate-inducible costimulatory pair that has been targeted for the alleviation of HSK is the 4–1BB/4–1BBL costimulatory signal [10]. Inhibition of the inducible 4–1BB/4–1BBL costimulatory pathway by administration of anti-mouse 4–1BBL mAb systemically inhibited CD4^+^ T-cell infiltration into infected corneas, resulting in reduced HSK [10].

BTLA, an inhibitory coreceptor that negatively regulates lymphocyte activation, plays a critical role in several immune-inflammatory diseases [18-20,30]. To determine whether BTLA takes part in the immunopathogenesis of HSK in BALB/c mice, we first tested the level of BTLA expression on CD4^+^ and CD8^+^ T cells in murine peripheral blood by flow cytometry during HSK. At the same time, we also confirmed BTLA expression in the murine cornea by immunohistochemistry. We found that BTLA expression increased both in the cornea of HSV-1 infected mice and in CD4^+^ T cells in the peripheral blood, indicating that BTLA does participate in the immune response that occurs in HSK.

The next issue was to investigate the role of BTLA during HSK. In this study, we investigated whether systemic administration of recombinant plasmid DNA encoding BTLA (pBTLA) could decrease the numbers of CD4^+^ T cells that infiltrated into infected corneas, resulting in reduced HSK. BALB/c mice that received an intraperitoneal administration of 100 µg of pBTLA on 0 and 7 days before infection and 7 days postinfection showed a diminished incidence and severity of the stromal disease. The absolute amount and percentage of CD4^+^ T cells from cornea of pBTLA treated mice were significantly lower than those of pcDNA3.1 treated mice or PBS control mice. The results indicated that systemic administration of pBTLA dramatically reduced CD4^+^ T cell response to HSV-1 infection in cornea. This could mean that BTLA-HVEM coinhibitory signaling may play a crucial role in preventing HSV-induced corneal immunopathology mediated by CD4^+^ T cells.

The role of pBTLA in CD4^+^ Th1 immune responses during HSK was further investigated by examining the number of CD4^+^ T cells expressing IFN-γ^+^ in the corneas of different group mice on day 14 after in vivo HSV-1 infection. The result shows that CD4^+^ T cells expressing IFN-γ^+^ in the cornea of pBTLA treated mice were fewer when compared with control groups. Flow cytometry measurement of IFN-γ^+^ T cells in the DLN from different group mice revealed similar results. We also collected splenocytes of different mice groups and tested HSV specific Th1 cell responses. The IFN-γ concentrations were reduced in splenocytes isolated from pBTLA treated mice when stimulated in vitro, indicating that pBTLA treated mice had a reduced Th1 immune response. This was also supported by our results for DTH responses in the mice.

The DTH response is an immune response that occurs when antigen activated Th1 cells release pro-inflammatory cytokines at the site of antigen recognition. In the eye, the cytokines, cellular infiltration, and vascular changes that accompany DTH responses are an immediate and long-term detriment to corneal clarity. In ocular HSV-1 infection, a reduction in the severity of HSK has been shown in DTH tolerant mice, while elevated DTH responses are associated with greater corneal pathology in HSK [23,31,32]. In the present study, pBTLA treated mice showed a reduced DTH response two weeks after HSV-1 infection compared to pcDNA or PBS treated mice. Systemic administration of recombinant pBTLA decreased the role of characteristic Th1 cytokines during HSV-1 infection.

The entry of HSV-1 into cells is well known to require membrane fusion between the virus envelope and cell membrane, which is primarily mediated by the interaction of the viral ligand, glycoprotein D(gD)and its cellular receptor HVEM [33-35]. HSV has evolved the ability to enter and infect T cells. Activation of T cells increased their permissivity toward HSV infection. HSV infection of both CD4^+^ and CD8^+^ T cells occurred much more efficiently via direct cell-to-cell spread from infected fibroblasts than by cell-free virus. Transfer of HSV to T cells depends mainly on the binding of gD to its receptor, HVEM [36]. gD is expressed on the surface of activated T cells after infection [37]. The membrane-bound gD can exert an immunomodulatory effect on activated T cells [35,37-39]. Comparison of the gD and BTLA binding sites on HVEM shows that the BTLA and gD binding sites are largely overlapping and involve similar structural motifs as well as similar energetics [12,40]. However, in this report we demonstrate that intraperitoneal application of recombinant pBTLA did not affect gD-mRNA levels or tear film virus titers in corneas. Further study will be done on the corneal treatment with pBTLA to test whether intraocular administration can affect corneal viral titer and gD levels.

In summary, the present study provides evidence that the BTLA-HVEM coinhibitory signal is functionally involved in the HSV-induced corneal immunopathology mediated by CD4^+^ T cells. Promoting the BTLA-HVEM interaction by recombinant pBTLA may represent an ideal target for HSK therapy to suppress the immune reaction and avoid corneal scarring.
